# Web-Based Software Tools for Systematic Literature Review in Medicine: Systematic Search and Feature Analysis

**DOI:** 10.2196/33219

**Published:** 2022-05-02

**Authors:** Kathryn Cowie, Asad Rahmatullah, Nicole Hardy, Karl Holub, Kevin Kallmes

**Affiliations:** 1 Nested Knowledge Saint Paul, MN United States

**Keywords:** software tools, feature analysis, systematic reviews

## Abstract

**Background:**

Systematic reviews (SRs) are central to evaluating therapies but have high costs in terms of both time and money. Many software tools exist to assist with SRs, but most tools do not support the full process, and transparency and replicability of SR depend on performing and presenting evidence according to established best practices.

**Objective:**

This study aims to provide a basis for comparing and selecting between web-based software tools that support SR, by conducting a feature-by-feature comparison of SR tools.

**Methods:**

We searched for SR tools by reviewing any such tool listed in the SR Toolbox, previous reviews of SR tools, and qualitative Google searching. We included all SR tools that were currently functional and required no coding, and excluded reference managers, desktop applications, and statistical software. The list of features to assess was populated by combining all features assessed in 4 previous reviews of SR tools; we also added 5 features (manual addition, screening automation, dual extraction, living review, and public outputs) that were independently noted as best practices or enhancements of transparency and replicability. Then, 2 reviewers assigned binary *present* or *absent* assessments to all SR tools with respect to all features, and a third reviewer adjudicated all disagreements.

**Results:**

Of the 53 SR tools found, 55% (29/53) were excluded, leaving 45% (24/53) for assessment. In total, 30 features were assessed across 6 classes, and the interobserver agreement was 86.46%. Giotto Compliance (27/30, 90%), DistillerSR (26/30, 87%), and Nested Knowledge (26/30, 87%) support the most features, followed by EPPI-Reviewer Web (25/30, 83%), LitStream (23/30, 77%), JBI SUMARI (21/30, 70%), and SRDB.PRO (VTS Software) (21/30, 70%). Fewer than half of all the features assessed are supported by 7 tools: RobotAnalyst (National Centre for Text Mining), SRDR (Agency for Healthcare Research and Quality), SyRF (Systematic Review Facility), Data Abstraction Assistant (Center for Evidence Synthesis in Health), SR Accelerator (Institute for Evidence-Based Healthcare), RobotReviewer (RobotReviewer), and COVID-NMA (COVID-NMA). Notably, of the 24 tools, only 10 (42%) support direct search, only 7 (29%) offer dual extraction, and only 13 (54%) offer living/updatable reviews.

**Conclusions:**

DistillerSR, Nested Knowledge, and EPPI-Reviewer Web each offer a high density of SR-focused web-based tools. By transparent comparison and discussion regarding SR tool functionality, the medical community can both choose among existing software offerings and note the areas of growth needed, most notably in the support of living reviews.

## Introduction

### Systematic Review Costs and Gaps

According to the Centre for Evidence-Based Medicine, systematic reviews (SRs) of high-quality primary studies represent the highest level of evidence for evaluating therapeutic performance [1]. However, although vital to evidence-based medical practice, SRs are time-intensive, taking an average of 67.3 weeks to complete [2] and costing leading research institutions over US $141,000 in labor per published review [3]. Owing to the high costs in researcher time and complexity, up-to-date reviews cover only 10% to 17% of primary evidence in a representative analysis of the lung cancer literature [4]. Although many qualitative and noncomprehensive publications provide some level of summative evidence, SRs—defined as reviews of “evidence on a clearly formulated question that use systematic and explicit methods to identify, select and critically appraise relevant primary research, and to extract and analyze data from the studies that are included” [5]—are distinguished by both their structured approach to finding, filtering, and extracting from underlying articles and the resulting comprehensiveness in answering a concrete medical question.

### Software Tools for Systematic Review

Software tools that assist with central SR activities—retrieval (searching or importing records), appraisal (screening of records), synthesis (content extraction from underlying studies), and documentation/output (presentation of SR outputs)—have shown promise in reducing the amount of effort needed in a given review [6]. Because of the time savings of web-based software tools, institutions and individual researchers engaged in evidence synthesis may benefit from using these tools in the review process [7].

### Existing Studies of Software Tools

However, choosing among the existing software tools presents a further challenge to researchers; in the SR Toolbox [8], there are >240 tools indexed, of which 224 support health care reviews. Vitally, few of these tools can be used for each of the steps of SR, so comparing the features available through each tool can assist researchers in selecting an SR tool to use. This selection can be informed by feature analysis; for example, a previously published feature analysis compared 15 SR tools [9] across 21 subfeatures of interest and found that DistillerSR (Evidence Partners), EPPI-Reviewer (EPPI-Centre), SWIFT-Active Screener (Sciome), and Covidence (Cochrane) support the greatest number of features as of 2019. Harrison et al [10], Marshall et al [11], and Kohl et al [12] have completed similar analyses, but each feature assessment selected a different set of features and used different qualitative feature assessment methods, and none covered all SR tools currently available.

The SR tool landscape continues to evolve; as existing tools are updated, new software is made available to researchers, and new feature classes are developed. For instance, despite the growth of calls for living SRs, that is, reviews where the outputs are updated as new primary evidence becomes available, no feature analysis has yet covered this novel capability. Furthermore, the leading feature analyses [9-12] have focused on the screening phase of review, meaning that no comparison of data extraction capabilities has yet been published.

### Feature Analysis of Systematic Review Tools

The authors, who are also the developers of the Nested Knowledge platform for SR and meta-analysis (Nested Knowledge, Inc) [13], have noted the lack of SR feature comparison among new tools and across all feature classes (retrieval, appraisal, synthesis, documentation/output, administration of reviews, and access/support features). To provide an updated feature analysis comparing SR software tools, we performed a feature analysis covering the full life cycle of SR across software tools.

## Methods

### Search Strategy

We searched the SR tools for assessment in 3 ways: first, we identified any SR tool that was published in existing reviews of SR tools (Table S1 in Multimedia Appendix 1). Second, we reviewed SR Toolbox [8], a repository of indexed software tools that support the SR process. Third, we performed a Google search for *Systematic review software* and identified any software tool that was among the first 5 pages of results. Furthermore, for any library resource pages that were among the search results, we included any SR tools mentioned by the library resource page that met our inclusion criteria. The search was completed between June and August 2021. Four additional tools, namely SRDR+ (Agency for Healthcare Research and Quality), Systematic Review Assistant-Deduplication Module (Institute for Evidence-Based Healthcare), Giotto Compliance, and Robotsearch (Robotsearch), were assessed in December 2021 following reviewer feedback.

### Selection of Software Tools

The inclusion and exclusion criteria were determined by 3 authors (KK, KH, and KC). Among our search results, we queued up all software tools that had descriptions meeting our inclusion criteria for full examination of the software in a second round of review. We included any that were functioning web-based tools that require no coding by the user to install or operate, so long as they were used to support the SR process and can be used to review clinical or preclinical literature. The *no coding* requirement was established because the target audience of this review is medical researchers who are selecting a review software to use; thus, we aim to review only tools that this broad audience is likely to be able to adopt. We also excluded desktop applications, statistical packages, and tools built for reviewing software engineering and social sciences literature, as well as reference managers, to avoid unfairly casting these tools as incomplete review tools (as they would each score quite low in features that are not related to reference management). All software tools were screened by one reviewer (KC), and inclusion decisions were reviewed by a second (KK).

### Selection of Features of Interest

We built on the previous comparisons of SR tools published by Van der Mierden et al [9], Harrison et al [10], Marshall et al [11], and Kohl et al [12], which assign features a level of importance and evaluate each feature in reference screening tools. As the studies by Van der Mierden et al [9] and Harrison et al [10] focus on reference screening, we supplemented the features with features identified in related reviews of SR tools (Table S1 in Multimedia Appendix 1). From a study by Kohl et al [12], we added database search, risk of bias assessment (critical appraisal), and data visualization. From Marshall et al [11], we added report writing.

We added 4 more features based on their importance to software-based SR: manual addition of records, automated full-text retrieval, dual extraction of studies, risk of bias (critical appraisal), living SR, and public outputs. Each addition represents either a best practice in SR [14] or a key feature for the accuracy, replicability, and transparency of SR. Thus, in total, we assessed the presence or absence of 30 features across 6 categories: retrieval, appraisal, synthesis, documentation/output, administration/project management, and access/support.

We adopted each feature unless it was outside of the SR process, it was required for inclusion in the present review, it duplicated another feature, it was not a discrete step for comparison, it was not necessary for English language reviews, it was not necessary for a web-based software, or it related to reference management (as we excluded reference managers from the present review). Table 1 shows all features not assessed, with rationale.

**Table 1:** Features from systematic reviews not assessed in this review, with rationale.

Features not assessed	Rationale
Functional	Part of our inclusion criteria
Reference allocation	Reference management excluded from this review
Randomizing order of references	Not part of systematic review process
Non-Latin character support	Review focused on English language systematic review software
Straightforward system requirements	Part of our inclusion criteria
Installation guide	Not necessary for web-based software
No coding	Part of our inclusion criteria
Mobile- or tablet-responsive interface	Not necessary for web-based software
Other stages	Not a discrete or comparable step
Multiple projects	Not part of the systematic review process
Work allocation	Duplicated with “distinct user roles”
Export of decisions	Duplicated with export
User setup	Duplicated with “distinct user roles”
Filter references	Duplicated with screening records
Search references	Duplicated with “database search”
Insecure website	Information not available to reviewers
Security	Information not available to reviewers
Setting up review	Not a discrete or comparable step
Automated analysis	Not a discrete or comparable step
Text analysis	Not part of the systematic review process
Report validation	Not part of the systematic review process
Document management	Reference management excluded from this review
Bibliography	Reference management excluded from this review

### Feature Assessment

To minimize bias concerning the subjective assessment of the necessity or desirability of features or of the relative performance of features, we used a binary assessment where each SR tool was scored 0 if a given feature was not present or 1 if a feature was present. Tools were assessed between June and August 2021. We assessed 30 features, divided into 6 feature classes. Of the 30 features, 77% (23/30) were identified in existing literature, and 23% (7/30) were added by the authors (Table 2).

**Table 2:** The criteria for each selected feature, as well as the rationale.

Classification and variable name and coding			Feature from	Rationale (if added by authors)
**Retrieval**				
	Database search	1—literature search through API^a^ Integration with a database; 0—no method for retrieving studies directly from a database	Kohl et al [12], Marshall et al [11]	—^b^
	Reference importing	1—import of references as RIS^c^ files or other file types; 0—references have to be entered manually	Harrison et al [10], Van der Mierden et al [9]	—
	Manual addition	1—add a reference by entering study metadata; 0—no method for adding individual references and gray literature	Added by the authors	Ability to add expert additions is called for by the PRISMA^d^ 2020 guidelines and checklist [14]
	Attaching full-text PDFs	1—ability to import or upload full-text PDFs associated with each study under review; 0—no method for importing full-text PDFs in the screening process	Harrison et al [10], Van der Mierden et al [9]	—
	Automated full-text retrieval	1—ability to fetch some or all full texts via API or other nonmanual method; 0—full texts must be uploaded manually, or full-text upload not supported	Added by the authors	Full texts are required for content extraction, and manual upload represents a major time investment by the user
**Appraisal**				
	Title/abstract screening	1—inclusion and exclusion by title and abstract only; 0—no system for inclusion and exclusion of references by title and abstract	Harrison et al [10], Van der Mierden et al [9]	—
	Full-text screening	1—a distinct full-text screening phase; 0—there is no full-text screening phase	Harrison et al [10], Van der Mierden et al [9]	—
	Dual screening and adjudication	1—choice for single or double screening and a method for resolving conflicts; 0—no ability to configure screening mode or no ability to resolve conflicts	Harrison et al [10], Van der Mierden et al [9]	—
	Keyword highlighting	1—abstract keywords are highlighted. Keywords can be user or AI^e^-determined; 0—No keyword highlighting is possible	Harrison et al [10], Van der Mierden et al [9]	—
	Machine learning/automation (screening)	1—has a form of machine learning or automation of the screening process; 0—does not support any form of machine learning or automation of the screening process	Added by the authors	Automated screening has been called for by the scientific community [15]
	Deduplication of references	1—automatically identifies duplicate references or marks potential duplicates for manual review; 0—has no mechanism for deduplication	Harrison et al [10], Kohl et al [12]	—
**Extraction**				
	Tagging references	1—ability to attach tags that reflect the content of underlying studies to specific references; 0—no means for attaching content-related tags to references	Van der Mierden et al [9], Kohl et al [12]	—
	Data extraction	1—facilitates extraction and storage of quantitative data into a form or template; 0—does not permit extraction and storage or quantitative data	Harrison et al [10], Kohl et al [12], Marshall et al [11]	—
	Dual extraction	1—ability for 2 independent reviewers to collect on each study and for a third person to adjudicate differences; 0—no ability to have independent extraction and adjudication	Added by the authors	Dual extraction improves the accuracy of data gathering [16]
	Risk of bias	1—supports critical appraisal of studies through risk of bias assessments; 0—no built-in features or templates to assess risk of bias	Kohl et al [12]	—
**Documentation/output**				
	Flow diagram creation	1—automated or semiautomated creation of PRISMA flow diagrams; 0—the tool cannot automatically provide a flow diagram meeting the PRISMA criteria	Van der Mierden et al [9]	—
	Manuscript writing	1—ability to write or edit a report or manuscript; 0—no ability to write or edit a report or manuscript	Marshall et al [11]	—
	Citation management	1—ability to insert citations based on stored study metadata into a text editor; 0—no ability to insert citations into a document	Added by the authors	The ability to add and manage citations is necessary to document the source of review data
	Data visualizations	1—generation of figures or tables to assist with data presentation; 0—no built-in way to generate figures or tables	Kohl et al [12]	—
	Export	1—supports export of references, study metadata, or collected data; 0—has no export feature	Harrison et al [10], Van der Mierden et al [9]	—
**Admin**				
	Protocol	1—supports protocol development or filling in a research question template; 0—no protocol development or templates	Kohl et al [12], Marshall et al [11]	—
	Distinct user roles	1—distinct user roles and permissions; 0—no distinct roles; everybody has the same role and rights in the project	Harrison et al [10], Van der Mierden et al [9], Marshall et al [11]	—
	Activity monitoring	1—software monitors and displays progress through the project; 0—there is no way to determine overall progress of the project (eg, % completed)	Harrison et al [10], Van der Mierden et al [9]	—
	Comments or chat	1—ability to leave comments or notes on studies; 0—it is not possible to attach comments to references	Van der Mierden et al [9]	—
	Training	1—there are publicly available web-based tutorials, help pages, training videos, or forums maintained by the software provider; 0—there are no accessible tutorials or training materials maintained by the software provider	Harrison et al [10], Marshall et al {11]	—
	Customer support	1—customer support, such as support contact information, is provided on request; 0—customer support is not clearly available	Van der Mierden et al [9]	—
**Access and support**				
	Pricing (free to use)	1—a free version is available for users; 0—the tool must be purchased, or free or trial accounts have severe limitations that can compromise the systematic review	Harrison et al [10], Van der Mierden et al [9], Marshall et al [11]	—
	Living/updatable	1—new records can be added after a project has been completed; 0—new records cannot be added after a project has been completed	Added by the authors	Living systematic review has been called for as a novel paradigm solving the main limitation of systematic review [17]
	Public outputs	1—web-based visualizations or writing can be made publicly visible; 0—review data and outputs cannot be made publicly visible	Added by the authors	Web-based availability of systematic review outputs is important for transparency and replicability of research [18]
	User collaboration	1—multiple users can work simultaneously on 1 review; 0—it is not possible for multiple users to work at the same time on the same project, independently	Harrison et al [10], Van der Mierden et al [9], Marshall et al [11]	—

^a^API: application programming interface.

^b^Rationale only provided for features added in this review; all other features were drawn from existing feature analyses of Systematic Review Software Tools.

^c^RIS: Research Information System.

^d^PRISMA: Preferred Reporting Items for Systematic Reviews and Meta-Analyses.

^e^AI: artificial intelligence.

### Evaluation of Tools

For tools with free versions available, each of the researchers created an account and tested the program to determine feature presence. We also referred to user guides, publications, and training tutorials. For proprietary software, we gathered information on feature offerings from marketing webpages, training materials, and video tutorials. We also contacted all proprietary software providers to give them the opportunity to comment on feature offerings that may have been left out of those materials. Of the 8 proprietary software providers contacted, 38% (3/8) did not respond, 50% (4/8) provided feedback on feature offerings, and 13% (1/8) declined to comment. When providers provided feedback, we re-reviewed the features in question and altered the assessment as appropriate. One provider gave feedback after initial puplication, prompting issuance of a correction.

Feature assessment was completed independently by 2 reviewers (KC and AR), and all disagreements were adjudicated by a third (KK). Interobserver agreement was calculated using standard methods [19] as applied to binary assessments. First, the 2 independent assessments were compared, and the number of disagreements was counted per feature, per software. For each feature, the total number of disagreements was counted and divided by the number of software tools assessed. This provided a per-feature variability percentage; these percentages were averaged across all features to provide a cumulative interobserver agreement percentage.

## Results

### Identification of SR Tools

**Figure 1:**
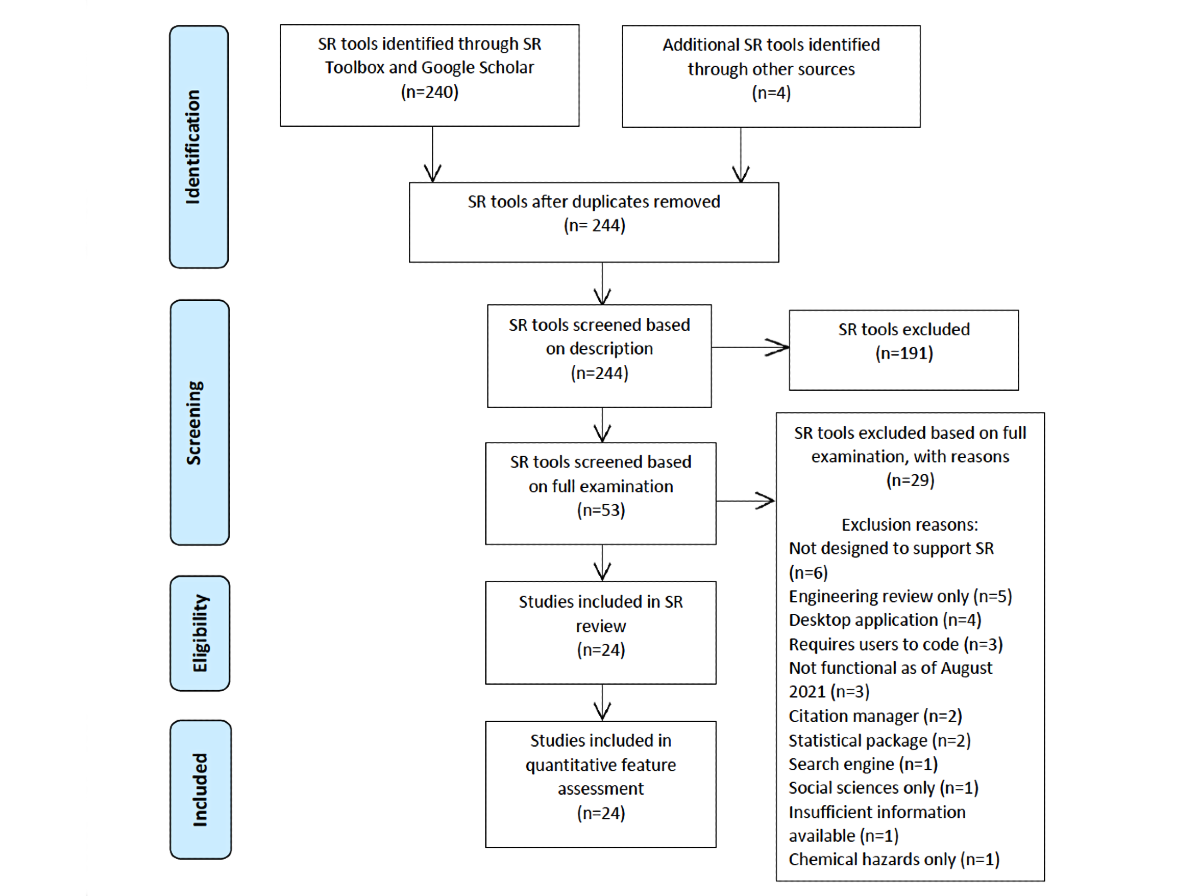
PRISMA (Preferred Reporting Items for Systematic Reviews and Meta-Analyses)-based chart showing the sources of all tools considered for inclusion, including 2-phase screening and reasons for all exclusions made at the full software review stage. SR: systematic review.

We reviewed all 240 software tools offered on SR Toolbox and sent forward all studies that, based on the software descriptions, could meet our inclusion criteria; we then added in all software tools found on Google Scholar. This strategy yielded 53 software tools that were reviewed in full (Figure 1 shows the PRISMA [Preferred Reporting Items for Systematic Reviews and Meta-Analyses]-based chart). Of these 53 software tools, 55% (29/53) were excluded. Of the 29 excluded tools, 17% (5/29) were built to review software engineering literature, 10% (3/29) were not functional as of August 2021, 7% (2/29) were citation managers, and 7% (2/29) were statistical packages. Other excluded tools included tools not designed for SRs (6/29, 21%), desktop applications (4/29, 14%), tools requiring users to code (3/29, 10%), a search engine (1/29, 3%), and a social science literature review tool (1/29, 3%). One tool, Research Screener [20], was excluded owing to insufficient information available on supported features. Another tool, the Health Assessment Workspace Collaborative, was excluded because it is designed to assess chemical hazards.

### Overview of SR Tools

We assessed the presence of features in 24 software tools, of which 71% (17/24) are designed for health care or biomedical sciences. In addition, 63% (15/24) of the analyzed tools support the full SR process, meaning they enable search, screening, extraction, and export, as these are the basic capabilities necessary to complete a review in a single software tool. Furthermore, 21% (5/34) of the tools support the screening stage (Table 3).

**Table 3:** Breakdown of software tools for systematic review by process type (full process, screening, extraction, or visualization; n=24).

Type	Tools, n (%)	Software tools
Full process	15 (63)	Cadima, Covidence, Colandr, DistillerSR, EPPI-Reviewer Web, Giotto Compliance, JBI SUMARI, LitStream, Nested Knowledge, PICOPortal, Revman Web, SRDB.PRO, SRDR+, SyRF, SysRev
Screening	5 (21)	Abstrackr, Rayyan, RobotAnalyst, SWIFT-Active Screener, SR Accelerator
Extraction	3 (13)	Data Abstraction Assistant, RobotReviewer, SRDR
Visualization	1 (4)	COVID-NMA

### Data Gathering

Interobserver agreement between the 2 reviewers gathering data features was 86.46%, meaning that across all feature assessments, the 2 reviewers disagreed on <15% of the applications. Final assessments are summarized in Table 4, and Table S2 in Multimedia Appendix 2 shows the interobserver agreement on a per–SR tool and per-feature basis. Interobserver agreement was ≥70% for every feature assessed and for all SR tools except 3: LitStream (ICF; 53.3%), RevMan Web (Cochrane; 50%), and SR Accelerator (Institute for Evidence-Based Healthcare; 53.3%); on investigation, these low rates of agreement were found to be due to name changes and versioning (LitStream and RevMan Web) and due to the modular nature of the subsidiary offerings (SR Accelerator). An interactive, updatable visualization of the features offered by each tool is available in the Systematic Review Methodologies Qualitative Synthesis.

**Table 4:** Feature assessment scores by feature class for each systematic review tool analyzed. The total number of features across all feature classes is presented in descending order.

Systematic review tool	Retrieval (n=5), n (%)	Appraisal (n=6), n (%)	Extraction (n=4), n (%)	Output (n=5), n (%)	Admin (n=6), n (%)	Access (n=4), n (%)	Total (n=30), n (%)
Giotto Compliance	5 (100)	6 (100)	4 (100)	3 (60)	6 (100)	3 (75)	27 (90)
DistillerSR	5 (100)	6 (100)	3 (75)	4 (80)	6 (100)	2 (50)	26 (87)
Nested Knowledge	4 (80)	5 (83)	2 (50)	5 (100)	6 (100)	4 (100)	26 (87)
EPPI-Reviewer Web	4 (80)	6 (100)	4 (100)	3 (60)	5 (83)	3 (75)	25 (83)
LitStream	2 (40)	5 (83)	3 (75)	3 (60)	6 (100)	4 (100)	23 (77)
JBI SUMARI	3 (60)	4 (67)	2 (50)	4 (80)	5 (83)	3 (75)	21 (70)
SRDB.PRO	5 (100)	4 (67)	2 (50)	3 (60)	6 (100)	1 (25)	21 (70)
Covidence	3 (60)	5 (83)	4 (100)	2 (40)	5 (83)	1 (25)	20 (67)
SysRev	4 (80)	3 (50)	2 (50)	2 (40)	5 (83)	4 (100)	20 (67)
Cadima	2 (40)	5 (83)	3 (75)	2 (40)	4 (67)	3 (75)	19 (63)
SRDR+	2 (40)	3 (50)	3 (75)	1 (20)	6 (100)	4 (100)	19 (63)
Colandr	4 (80)	6 (100)	1 (25)	2 (40)	3 (50)	2 (50)	18 (60)
PICOPortal	2 (40)	6 (100)	2 (50)	2 (40)	3 (50)	3 (75)	18 (60)
Rayyan	3 (60)	5 (83)	2 (50)	2 (40)	4 (50)	2 (50)	18 (60)
Revman Web	2 (40)	1 (17)	2 (50)	3 (60)	6 (100)	3 (75)	17 (57)
SWIFT-Active Screener	3 (60)	6 (100)	0 (0)	1 (20)	5 (83)	1 (25)	16 (53)
Abstrackr	1 (20)	5 (83)	1 (25)	1 (20)	5 (83)	2 (50)	15 (50)
RobotAnalyst	2 (40)	3 (50)	0 (0)	2 (40)	5 (83)	2 (50)	14 (47)
SRDR	1 (20)	0 (0)	2 (50)	2 (40)	5 (83)	4 (100)	14 (47)
SyRF	1 (20)	4 (67)	2 (50)	1 (20)	2 (33)	2 (50)	12 (40)
Data Abstraction Assistant	2 (40)	0 (0)	1 (25)	0 (0)	3 (50)	4 (100)	10 (33)
SR-Accelerator	2 (40)	4 (67)	0 (0)	0 (0)	2 (33)	1 (25)	9 (30)
RobotReviewer	2 (40)	0 (0)	2 (50)	1 (20)	2 (33)	1 (25)	8 (27)
COVID-NMA	0 (0)	0 (0)	0 (0)	2 (40)	1 (17)	3 (75)	6 (20)

### Feature Assessment

Giotto Compliance (27/30, 90%), DistillerSR (26/30, 87%), and Nested Knowledge (26/30, 87%) support the most features, followed by EPPI-Reviewer Web (25/30, 83%), LitStream (23/30, 77%), JBI SUMARI (21/30, 70%), and SRDB.PRO (VTS Software) (21/30, 70%).

**Figure 2:**
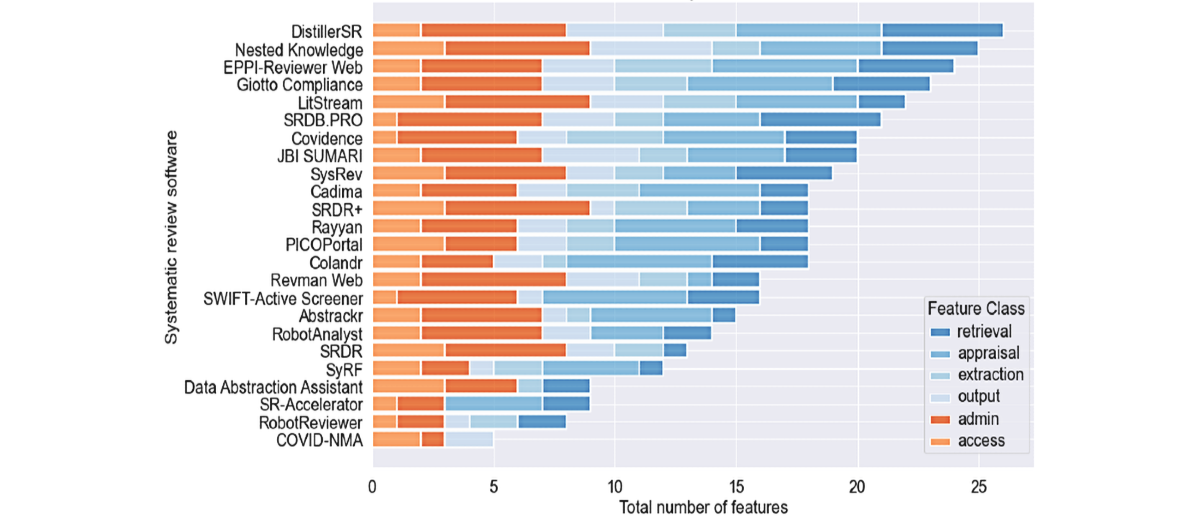
Stacked bar chart comparing the percentage of supported features, broken down by their feature class (retrieval, appraisal, extraction, output, admin, and access), among all analyzed software tools.

The top 16 software tools are ranked by percent of features from highest to lowest in Figure 2. Fewer than half of all features are supported by 7 tools: RobotAnalyst (National Centre for Text Mining), SRDR (Agency for Healthcare Research and Quality), SyRF (Systematic Review Facility), Data Abstraction Assistant (Center for Evidence Synthesis in Health, Institute for Evidence-Based Healthcare), SR-Accelerator, RobotReviewer (RobotReviewer), and COVID-NMA (COVID-NMA; Table 3).

### Feature Assessment: Breakout by Feature Class

**Figure 3:**
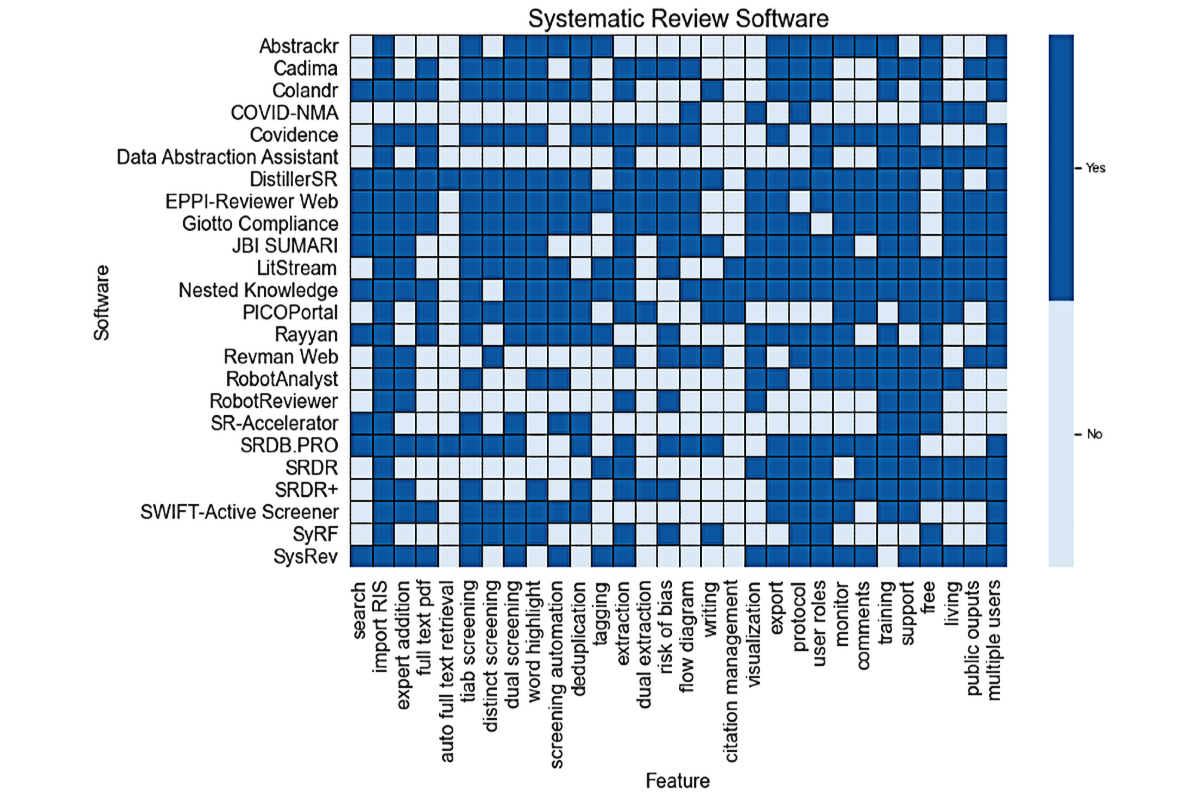
Heat map of features observed in 24 analyzed software tools. Dark blue indicates that a feature is present, and light blue indicates that a feature is not present.

Of all 6 feature classes, administrative features are the most supported, and output and extraction features are the least supported (Figure 3). Only 3 tools, Covidence (Cochrane), EPPI-Reviewer, and Giotto Compliance, offer all 4 extraction features (Table 4). DistillerSR and Giotto support all 5 retrieval features, while Nested Knowledge supports all 5 documentation/output features. Colandr, DistillerSR, EPPI-Reviewer, Giotto Compliance, and PICOPortal support all 6 appraisal features.

#### Feature Class 1: Retrieval

The ability to search directly within the SR tool was only present for 42% (10/24) of the software tools, meaning that for all other SR tools, the user is required to search externally and import records. The only SR tool that did not enable importing of records was COVID-NMA, which supplies studies directly from the providers of the tool but does not enable the user to do so.

#### Feature Class 2: Appraisal

Among the 19 tools that have title/abstract screening, all tools except for RobotAnalyst and SRDR+ enable dual screening and adjudication. Reference deduplication is less widespread, with 58% (14/24) of the tools supporting it. A form of machine learning/automation during the screening stage is present in 54% (13/24) of the tools.

#### Feature Class 3: Extraction

Although 75% (18/24) of the tools offer data extraction, only 29% (7/24) offer dual data extraction (Giotto Compliance, DistillerSR, SRDR+, Cadima [Cadima], Covidence, EPPI-Reviewer, and PICOPortal [PICOPortal]). A total of 54% (13/24) of the tools enable risk of bias assessments.

#### Feature Class 4: Output

Exporting references or collected data is available in 71% (17/24) of the tools. Of the 24 tools, 54% (13/24) generate figures or tables, 42% (10/24) of tools generate PRISMA flow diagrams, 32% (8/24%) have report writing, and only 13% (3/34) have in-text citations.

#### Feature Class 5: Admin

Protocols, customer support, and training materials are available in 71% (17/24), 79% (19/24), and 83% (20/24) of the tools, respectively. Of all administrative features, the least well developed are progress/activity monitoring, which is offered 67% (16/24) of the tools, and comments, which are available in 58% (14/24) of the tools.

#### Feature Class 6: Access

Access features cover both collaboration during the review, cost, and availability of outputs. Of the 24 software tools, 83% (20/24) permit collaboration by allowing multiple users to work on a project. COVID-NMA, RobotAnalyst, RobotReviewer, and SR-Accelerator do not allow multiple users. In addition, of the 24 tools, 71% (17/24) offer a free subscription, whereas 29% (7/24) require paid subscriptions or licenses (Covidence, DistillerSR, EPPI-Reviewer Web, Giotto Compliance, JBI Sumari, SRDB.PRO, and SWIFT-Active Screener). Only 54% (13/24) of the software tools support living, updatable reviews.

## Discussion

### Principal Findings

Our review found a wide range of options in the SR software space; however, among these tools, many lacked features that are either crucial to the completion of a review or recommended as best practices. Only 63% (15/24) of the SR tools covered the full process from search/import through to extraction and export. Among these 15 tools, only 67% (10/15) had a search functionality directly built in, and only 47% (7/15) offered dual data extraction (which is the gold standard in quality control). Notable strengths across the field include collaborative mechanisms (offered by 20/24, 83% tools) and easy, free access (17/24, 71% of tools are free). Indeed, the top 4 software tools in terms of number of features offered (Giotto Compliance, DistillerSR, Nested Knowledge, and EPPI-Reviewer all offered between 83% and 90% of the features assessed. However, major remaining gaps include a lack of automation of any step other than screening (automated screening offered by 13/24, 54% of tools) and underprovision of living, updatable outputs.

### Major Gaps in the Provision of SR Tools

#### Search

Marshall et al [11] have previously noted that “the user should be able to perform an automated search from within the tool which should identify duplicate papers and handle them accordingly” [11]. Less than a third of tools (7/24, 29%) support search, reference import, and manual reference addition.

#### Study Selection

Screening of references is the most commonly offered feature and has the strongest offerings across features. All software tools that offer screening also support dual screening (with the exception of RobotAnalyst and SRDR+). This demonstrates adherence to SR best practices during the screening stage.

#### Automation and Machine Learning

Automation in medical SR screening has been growing. Some form of machine learning or other automation for screening literature is present in over half (13/24, 54%) of all the tools analyzed. Machine learning/screening includes reordering references, topic modeling, and predicting inclusion rates.

#### Data Extraction

In contrast to screening, extraction is underdeveloped. Although extraction is offered by 75% (18/24) tools, few tools adhere to SR best practices of dual extraction. This is a deep problem in the methods of review, as the error rate for manual extraction without dual extraction is highly variable and has even reached 50% in independent tests [16].

Although single extraction continues to be the only commonly offered method, the scientific community has noted that automating extraction would have value in both time savings and improved accuracy, but the field is as of yet underdeveloped. To quote a recent review on the subject of automated extraction, “[automation] techniques have not been fully utilized to fully or even partially automate the data extraction step of systematic review” [21]. The technologies to automate extraction have not achieved partial extraction at a sufficiently high accuracy level to be adopted; therefore, dual extraction is a pressing software requirement that is unlikely to be surpassed in the near future.

#### Project Management

Administrative features are well supported by SR software. However, there is a need for improved monitoring of review progress. Project monitoring is offered by 67% (16/24) of the tools, which is among the lowest of all admin features and likely the feature most closely associated with the quality of the outputs. As collaborative access is common and highly prized, SR software providers should recognize the barriers to collaboration in medical research; lack of mutual awareness, inertia in communication, and time management and capacity constraints are among the leading reasons for failure in interinstitutional research [22]. Project monitoring tools could assist with each of these pain points and improve the transparency and accountability within the research team.

#### Living Reviews

The scientific community has made consistent demands for SR processes to be rendered updatable, with the goal of improving the quality of evidence available to clinicians, health policymakers, and the medical public [23,24]. Despite these ongoing calls for change, living, updatable reviews are not yet standard in SR software tools. Only 54% (13/24) of the tools support living reviews, largely because living review depends on providing updatability at each step up through to outputs. However, until greater provision of living review tools is achieved, reviews will continue to fall out of date and out of sync with clinical practice [24].

### Study Limitations

In our study design, we elected to use a binary assessment, which limited the bias induced by the subjective appeal of any given tool. Therefore, these assessments did not include any comparison of quality or usability among the SR tools. This also meant that we did not use the Desmet [25] method, which ranks features by level of importance. We also excluded certain assessments that may impact user choices such as language translation features or translated training documentation, which is supported by some technologies, including DistillerSR. We completed the review in August 2021 but added several software tools following reviewer feedback; by adding *expert additions* without repeating the entire search strategy, we may have missed SR tools that launched between August and December 2021. Finally, the authors of this study are the designers of one of the leading SR tools, Nested Knowledge, which may have led to tacit bias toward this tool as part of the comparison.

By assessing features offered by web-based SR applications, we have identified gaps in current technologies and areas in need of development. Feature count does not equate to value or usability; it fails to capture benefits of simple platforms, such as ease of use, effective user interface, alignment with established workflows, or relative costs. The authors make no claim about superiority of software based on feature prevalence.

### Future Directions

We invite and encourage independent researchers to assess the landscape of SR tools and build on this review. We expect the list of features to be assessed will evolve as research changes. For example, this review did not include features such as the ability to search included studies, reuse of extracted data, and application programming interface calls to read data, which may grow in importance. Furthermore, this review assessed the presence of automation at a high level without evaluating details. A future direction might be characterizing specific types of automation models used in screening, as well as in other stages, for software applications that support SR of biomedical research.

### Conclusions

The highest-performing SR tools were DistillerSR, EPPI-Reviewer Web, and Nested Knowledge, each of which offer >80% of features. The most commonly offered and robust feature class was screening, whereas extraction (especially quality-controlled dual extraction) was underprovided. Living reviews, although strongly advocated for in the scientific community, were similarly underprovided by the SR tools reviewed here. This review enables the medical community to complete transparent and comprehensive comparison of SR tools and may also be used to identify gaps in technology for further development by the providers of these or novel SR tools.

### Disclaimer

This review of web-based software review software tools represents an attempt to best capture information from software providers’ websites, free trials, peer-reviewed publications, training materials, or software tutorials. The review is based primarily on publicly available information and may not accurately reflect feature offerings, as relevant information was not always available or clear to interpret. This evaluation does not represent the views or opinions of any of the software developers or service providers, except those of the authors. The review was completed in August 2021, and readers should refer to the respective software providers’ websites to obtain updated information on feature offerings.

## Appendix

Multimedia Appendix 1 Supplementary Table 1: Screening Decisions for SR (systematic review) Tools Reviewed in Full.

Multimedia Appendix 2 Supplementary Table 2: Inter-observer Agreement across (1) Systematic Review (SR) Tools and (2) Features Assessed.

## Abbreviations

PRISMA: Preferred Reporting Items for Systematic Reviews and Meta-Analyses
SR: systematic review
