# Correction: Web-Based Software Tools for Systematic Literature Review in Medicine: Systematic Search and Feature Analysis

**DOI:** 10.2196/43520

**Published:** 2022-11-23

**Authors:** Kathryn Cowie, Asad Rahmatullah, Nicole Hardy, Karl Holub, Kevin Kallmes

**Affiliations:** 1 Nested Knowledge Saint Paul, MN United States

In “Web-Based Software Tools for Systematic Literature Review in Medicine: Systematic Search and Feature Analysis” (JMIR Med Inform 2022;10(5):e33219) the authors noted some errors and made the following corrections:

1. For the “Access” category in Table 4, features included free, living, public outputs, and multiple users. In the originally published article, the feature "public outputs" was not counted, understating the total features offered. Therefore, Table 4 has been revised, as follows:

**Table 4:** Feature assessment scores by feature class for each systematic review tool analyzed. The total number of features across all feature classes is presented in descending order.

Systematic review tool	Retrieval (n=5), n (%)	Appraisal (n=6), n (%)	Extraction (n=4), n (%)	Output (n=5), n (%)	Admin (n=6), n (%)	Access (n=4), n (%)	Total (n=30), n (%)
Giotto Compliance	5 (100)	6 (100)	4 (100)	3 (60)	6 (100)	3 (75)	27 (90)
DistillerSR	5 (100)	6 (100)	3 (75)	4 (80)	6 (100)	2 (50)	26 (87)
Nested Knowledge	4 (80)	5 (83)	2 (50)	5 (100)	6 (100)	4 (100)	26 (87)
EPPI-Reviewer Web	4 (80)	6 (100)	4 (100)	3 (60)	5 (83)	3 (75)	25 (83)
LitStream	2 (40)	5 (83)	3 (75)	3 (60)	6 (100)	4 (100)	23 (77)
JBI SUMARI	3 (60)	4 (67)	2 (50)	4 (80)	5 (83)	3 (75)	21 (70)
SRDB.PRO	5 (100)	4 (67)	2 (50)	3 (60)	6 (100)	1 (25)	21 (70)
Covidence	3 (60)	5 (83)	4 (100)	2 (40)	5 (83)	1 (25)	20 (67)
SysRev	4 (80)	3 (50)	2 (50)	2 (40)	5 (83)	4 (100)	20 (67)
Cadima	2 (40)	5 (83)	3 (75)	2 (40)	4 (67)	3 (75)	19 (63)
SRDR+	2 (40)	3 (50)	3 (75)	1 (20)	6 (100)	4 (100)	19 (63)
Colandr	4 (80)	6 (100)	1 (25)	2 (40)	3 (50)	2 (50)	18 (60)
PICOPortal	2 (40)	6 (100)	2 (50)	2 (40)	3 (50)	3 (75)	18 (60)
Rayyan	3 (60)	5 (83)	2 (50)	2 (40)	4 (50)	2 (50)	18 (60)
Revman Web	2 (40)	1 (17)	2 (50)	3 (60)	6 (100)	3 (75)	17 (57)
SWIFT-Active Screener	3 (60)	6 (100)	0 (0)	1 (20)	5 (83)	1 (25)	16 (53)
Abstrackr	1 (20)	5 (83)	1 (25)	1 (20)	5 (83)	2 (50)	15 (50)
RobotAnalyst	2 (40)	3 (50)	0 (0)	2 (40)	5 (83)	2 (50)	14 (47)
SRDR	1 (20)	0 (0)	2 (50)	2 (40)	5 (83)	4 (100)	14 (47)
SyRF	1 (20)	4 (67)	2 (50)	1 (20)	2 (33)	2 (50)	12 (40)
Data Abstraction Assistant	2 (40)	0 (0)	1 (25)	0 (0)	3 (50)	4 (100)	10 (33)
SR-Accelerator	2 (40)	4 (67)	0 (0)	0 (0)	2 (33)	1 (25)	9 (30)
RobotReviewer	2 (40)	0 (0)	2 (50)	1 (20)	2 (33)	1 (25)	8 (27)
COVID-NMA	0 (0)	0 (0)	0 (0)	2 (40)	1 (17)	3 (75)	6 (20)

The originally published Table 4 can be found in Multimedia Appendix 1.

Accordingly, the in-text references to Table 4 were revised in the article, as follows:

2. In the originally published article, in the Abstract, the section "Results" was the following:

Of the 53 SR tools found, 55% (29/53) were excluded, leaving 45% (24/53) for assessment. In total, 30 features were assessed across 6 classes, and the interobserver agreement was 86.46%. DistillerSR (Evidence Partners; 26/30, 87%), Nested Knowledge (Nested Knowledge; 25/30, 83%), and EPPI-Reviewer Web (EPPI-Centre; 24/30, 80%) support the most features followed by Giotto Compliance (Giotto Compliance; 23/30, 77%), LitStream (ICF), and SRDB.PRO (VTS Software). Fewer than half of all the features assessed are supported by 7 tools: RobotAnalyst (National Centre for Text Mining), SRDR (Agency for Healthcare Research and Quality), SyRF (Systematic Review Facility), Data Abstraction Assistant (Center for Evidence Synthesis in Health), SR Accelerator (Institute for Evidence-Based Healthcare), RobotReviewer (RobotReviewer), and COVID-NMA (COVID-NMA). Notably, of the 24 tools, only 10 (42%) support direct search, only 7 (29%) offer dual extraction, and only 13 (54%) offer living/updatable reviews.

In the Abstract, the section "Results" has been revised, as follows:

Of the 53 SR tools found, 55% (29/53) were excluded, leaving 45% (24/53) for assessment. In total, 30 features were assessed across 6 classes, and the interobserver agreement was 86.46%. Giotto Compliance (27/30, 90%), DistillerSR (26/30, 87%), and Nested Knowledge (26/30, 87%) support the most features, followed by EPPI-Reviewer Web (25/30, 83%), LitStream (23/30, 77%), JBI SUMARI (21/30, 70%), and SRDB.PRO (VTS Software) (21/30, 70%). Fewer than half of all the features assessed are supported by 7 tools: RobotAnalyst (National Centre for Text Mining), SRDR (Agency for Healthcare Research and Quality), SyRF (Systematic Review Facility), Data Abstraction Assistant (Center for Evidence Synthesis in Health), SR Accelerator (Institute for Evidence-Based Healthcare), RobotReviewer (RobotReviewer), and COVID-NMA (COVID-NMA). Notably, of the 24 tools, only 10 (42%) support direct search, only 7 (29%) offer dual extraction, and only 13 (54%) offer living/updatable reviews.

3. In the originally published article, under Methods, the first paragraph of the section “Evaluation of Tools” was the following:

For tools with free versions available, each of the researchers created an account and tested the program to determine feature presence. We also referred to user guides, publications, and training tutorials. For proprietary software, we gathered information on feature offerings from marketing webpages, training materials, and video tutorials. We also contacted all proprietary software providers to give them the opportunity to comment on feature offerings that may have been left out of those materials. Of the 8 proprietary software providers contacted, 50% (4/8) did not respond, 38% (3/8) provided feedback on feature offerings, and 13% (1/8) declined to comment. When providers provided feedback, we re-reviewed the features in question and altered the assessment as appropriate.

The first paragraph of the section “Evaluation of Tools” has been revised, as follows:

For tools with free versions available, each of the researchers created an account and tested the program to determine feature presence. We also referred to user guides, publications, and training tutorials. For proprietary software, we gathered information on feature offerings from marketing webpages, training materials, and video tutorials. We also contacted all proprietary software providers to give them the opportunity to comment on feature offerings that may have been left out of those materials. Of the 8 proprietary software providers contacted, 38% (3/8) did not respond, 50% (4/8) provided feedback on feature offerings, and 13% (1/8) declined to comment. When providers provided feedback, we re-reviewed the features in question and altered the assessment as appropriate. One provider gave feedback after initial puplication, prompting issuance of a correction.

4. In the originally published article, under Results, the section "Feature Assessment" was the following:

DistillerSR (26/30, 87%), Nested Knowledge (25/30, 83%), and EPPI-Reviewer Web (24/30, 80%) support the most features, followed by Giotto Compliance (23/30, 77%), LitStream, and SRDB.PRO (VTS Software). The top 16 software tools are ranked by percent of features from highest to lowest in Figure 2. Fewer than half of all features are supported by 5 tools: RobotAnalyst (National Centre for Text Mining), SRDR (Agency for Healthcare Research and Quality), SyRF (Systematic Review Facility), Data Abstraction Assistant (Center for Evidence Synthesis in Health, Institute for Evidence-Based Healthcare), RobotReviewer (RobotReviewer), and COVID-NMA (COVID-NMA; Table 3).

The section “Feature Assessment” has been replaced, as follows:

Giotto Compliance (27/30, 90%), DistillerSR (26/30, 87%), and Nested Knowledge (26/30, 87%) support the most features, followed by EPPI-Reviewer Web (25/30, 83%), LitStream (23/30, 77%), JBI SUMARI (21/30, 70%), and SRDB.PRO (VTS Software) (21/30, 70%).

The top 16 software tools are ranked by percent of features from highest to lowest in Figure 2. Fewer than half of all features are supported by 7 tools: RobotAnalyst (National Centre for Text Mining), SRDR (Agency for Healthcare Research and Quality), SyRF (Systematic Review Facility), Data Abstraction Assistant (Center for Evidence Synthesis in Health, Institute for Evidence-Based Healthcare), SR-Accelerator, RobotReviewer (RobotReviewer), and COVID-NMA (COVID-NMA; Table 3).

5. In the originally published article, the section "Feature Assessment: Breakout by Feature Class" was the following:

Of all 6 feature classes, administrative features are the most supported, and extraction features are the least supported (Figure 3). Only 2 tools, Covidence (Cochrane) and EPPI-Reviewer, offer all 4 extraction features (Table 4). DistillerSR, Nested Knowledge, and JBI SUMARI (JBI) support all 4 documentation/output features.

The section “Feature Assessment: Breakout by Feature Class” has been revised, as follows:

Of all 6 feature classes, administrative features are the most supported, and output and extraction features are the least supported (Figure 3). Only 3 tools, Covidence (Cochrane), EPPI-Reviewer, and Giotto Compliance, offer all 4 extraction features (Table 4). DistillerSR and Giotto support all 5 retrieval features, while Nested Knowledge supports all 5 documentation/output features. Colandr, DistillerSR, EPPI-Reviewer, Giotto Compliance, and PICOPortal support all 6 appraisal features.

6. In the originally published article, under Discussion, the “Principal Findings” section was the following:

Our review found a wide range of options in the SR software space; however, among these tools, many lacked features that are either crucial to the completion of a review or recommended as best practices. Only 63% (15/24) of the SR tools covered the full process from search/import through to extraction and export. Among these 15 tools, only 67% (10/15) had a search functionality directly built in, and only 47% (7/15) offered dual data extraction (which is the gold standard in quality control). Notable strengths across the field include collaborative mechanisms (offered by 20/24, 83% tools) and easy, free access (17/24, 71% of tools are free). Indeed, the top 4 software tools in terms of number of features offered (DistillerSR, Nested Knowledge, EPPI-Reviewer, and Giotto Compliance) all offered between 80% and 87% of the features assessed. However, major remaining gaps include a lack of automation of any step other than screening (automated screening offered by 13/24, 54% of tools) and underprovision of living, updatable outputs.

The section “Principal Findings” has been revised, as follows:

Our review found a wide range of options in the SR software space; however, among these tools, many lacked features that are either crucial to the completion of a review or recommended as best practices. Only 63% (15/24) of the SR tools covered the full process from search/import through to extraction and export. Among these 15 tools, only 67% (10/15) had a search functionality directly built in, and only 47% (7/15) offered dual data extraction (which is the gold standard in quality control). Notable strengths across the field include collaborative mechanisms (offered by 20/24, 83% tools) and easy, free access (17/24, 71% of tools are free). Indeed, the top 4 software tools in terms of number of features offered (Giotto Compliance, DistillerSR, Nested Knowledge, and EPPI-Reviewer all offered between 83% and 90% of the features assessed. However, major remaining gaps include a lack of automation of any step other than screening (automated screening offered by 13/24, 54% of tools) and underprovision of living, updatable outputs.

The authors confirm that these data changes do not affect the conclusions of the paper.

The correction will appear in the online version of the paper on the JMIR Publications website on November 23, 2022, together with the publication of this correction notice. Because this was made after submission to PubMed, PubMed Central, and other full-text repositories, the corrected article has also been resubmitted to those repositories.

## Appendix

Multimedia Appendix 1 Original Table 4.

