# Supplementary material for: Web-Based Software Tools for Systematic Literature Review in Medicine: Systematic Search and Feature Analysis
Source: JMIR Med Inform. 2022 May 2;10(5):e33219. doi: 10.2196/33219 (PMC9112080; doi:10.2196/33219)
Supplement: Multimedia Appendix 1 [file medinform_v10i4e33219_app1.docx]

**Supplementary Table 1:** Screening Decisions for Systematic Review Tools Reviewed in Full

| **NAME** | **TYPE** | **FIELD** | **STATUS** | **EXCLUSION REASON** |
| --- | --- | --- | --- | --- |
| *3d-NMA* | visualization | healthcare | excluded | not functional |
| ASReview | screening |  | excluded | requires user to code |
| Buhos |  |  | excluded | social sciences |
| Endnote | screening |  | excluded | not designed to support SR |
| EROS | screening | healthcare | excluded | not functional |
| Excel |  |  | excluded | not designed to support SR |
| HAWC Health Assessment Workspace Collaborative | systematic review |  | excluded | environmental health assessments of chemicals |
| Mendeley | citation manager |  | excluded | citation manager |
| Metagear | statistics |  | excluded | statistical package |
| Microsoft Word | document editor |  | excluded | not designed to support SR |
| PARSIFAL |  |  | excluded | software engineering tool |
| ReLiS (Revue Litteraire Systématique) |  |  | excluded | requires user to code |
| Research Screener |  |  | excluded | insufficient information available |
| REviewER |  |  | excluded | software engineering tool |
| Revman 5 |  |  | excluded | desktop application |
| RevManHAL | add on program for writing |  | excluded | desktop application |
| revtools | R package with deduplication |  | excluded | statistical package |
| RobotSearch | Search Tool for RCTs |  | excluded | not designed to support SR |
| SESRA |  |  | excluded | software engineering tool |
| SLR Tool (Middlesex) |  |  | excluded | not functional |
| SLR-tool |  |  | excluded | requires user to code |
| SLuRp (systematic literature unifed Review program) |  |  | excluded | software engineering tool |
| SRA-Helper for EndNote |  |  | excluded | desktop application |
| StArt (state of the art through systematic review) |  |  | excluded | software engineering tool |
| Stat59 | statistical program |  | excluded | not designed to support SR |
| *SWIFT-Review* | search and extraction | multidiscipline | excluded | desktop application |
| Systematic Review Assistant-Deduplication Module | Deduplication |  | excluded | not designed to support SR |
| TRIP Database | search engine |  | excluded | search engine only |
| Zotero | citation manager |  | excluded | citation manager |
| Abstrackr | screening | healthcare | included | N/A |
| Cadima | full process | multidiscipline | included | N/A |
| Colandr | full process | multidiscipline | included | N/A |
| COVID-NMA | visualization | healthcare | included | N/A |
| Covidence | full process | healthcare | included | N/A |
| Data Abstraction Assistant | extraction | healthcare | included | N/A |
| DistillerSR | full process | healthcare | included | N/A |
| EPPI-Reviewer Web | full process | healthcare | included | N/A |
| Giotto Compliance | full process | healthcare | included | N/A |
| JBI SUMARI | full process | multidiscipline | included | N/A |
| LitStream | full process | multidiscipline | included | N/A |
| Nested Knowledge | full process | healthcare | included | N/A |
| PICOPortal | full process | healthcare | included | N/A |
| Rayyan | screening | healthcare | included | N/A |
| Revman Web | full process | healthcare | included | N/A |
| RobotAnalyst | screening | healthcare | included | N/A |
| *RobotReviewer* | extraction | healthcare | included | N/A |
| SR-Accelerator | search and screening | multidiscipline | included | N/A |
| SRDB.PRO | full process | healthcare | included | N/A |
| SRDR | extraction | healthcare | included | N/A |
| SRDR+ | full process | healthcare | included | N/A |
| SWIFT-Active Screener | screening | multidiscipline | included | N/A |
| SyRF | full process | healthcare | included | N/A |
| SysRev | full process | multidiscipline | included | N/A |
