# Supplementary material for: Web-Based Software Tools for Systematic Literature Review in Medicine: Systematic Search and Feature Analysis
Source: JMIR Med Inform. 2022 May 2;10(5):e33219. doi: 10.2196/33219 (PMC9112080; doi:10.2196/33219)
Supplement: Multimedia Appendix 2 [file medinform_v10i4e33219_app2.docx]

| **SR Tool** | **Inter-observer Agreement (across 30 features)** | **Feature** | **Inter-observer Agreement (across 24 software tools)** |
| --- | --- | --- | --- |
| Abstrackr | 100.00% | search | 87.50% |
| Cadima | 100.00% | import | 87.50% |
| Colandr | 96.67% | expertaddition | 75.00% |
| COVID-NMA | 76.67% | tiabscreen | 91.67% |
| Covidence | 96.67% | distinctscreen | 83.33% |
| Data Abstraction Assistant | 90.00% | dualscreen | 91.67% |
| DistillerSR | 93.33% | fulltextpdf | 83.33% |
| EPPI-Reviewer Web | 96.67% | wordhighlight | 95.83% |
| Giotto Compliance | 70.00% | screeninautomation | 91.67% |
| JBI SUMARI | 93.33% | deduplication | 87.50% |
| LitStream | 53.33% | AutoFulltext | 95.83% |
| Nested Knowledge | 90.00% | tag | 91.67% |
| PicoPortal | 90.00% | extract | 91.67% |
| Rayyan | 93.33% | dualextract | 70.83% |
| Revman Web | 50.00% | riskofbias | 100.00% |
| RobotAnalyst | 96.67% | flowdiagram | 79.17% |
| RobotReviewer | 70.00% | writing | 79.17% |
| SR-Accelerator | 53.33% | citationmanager | 87.50% |
| SRDB.PRO | 90.00% | visualization | 83.33% |
| SRDR | 100.00% | export | 83.33% |
| SRDR+ | 90.00% | protocol | 87.50% |
| SWIFT-Active Screener | 96.67% | userroles | 91.67% |
| SyRF | 100.00% | monitor | 83.33% |
| SysRev | 70.00% | multipleusers | 83.33% |
| - | - | comments | 83.33% |
| - | - | free | 95.83% |
| - | - | living | 83.33% |
| - | - | training | 70.83% |
| - | - | support | 79.17% |
| - | - | publicoutputs | 75.00% |

**Supplementary Table 2:** Inter-observer Agreement across (1) Systematic Review (SR) Tools and (2) Features assessed.
