# Supplementary figures and images for: Correction: Web-Based Software Tools for Systematic Literature Review in Medicine: Systematic Search and Feature Analysis
Source: JMIR Med Inform. 2022 Nov 23;10(11):e43520. doi: 10.2196/43520 (PMC9746781; doi:10.2196/43520)

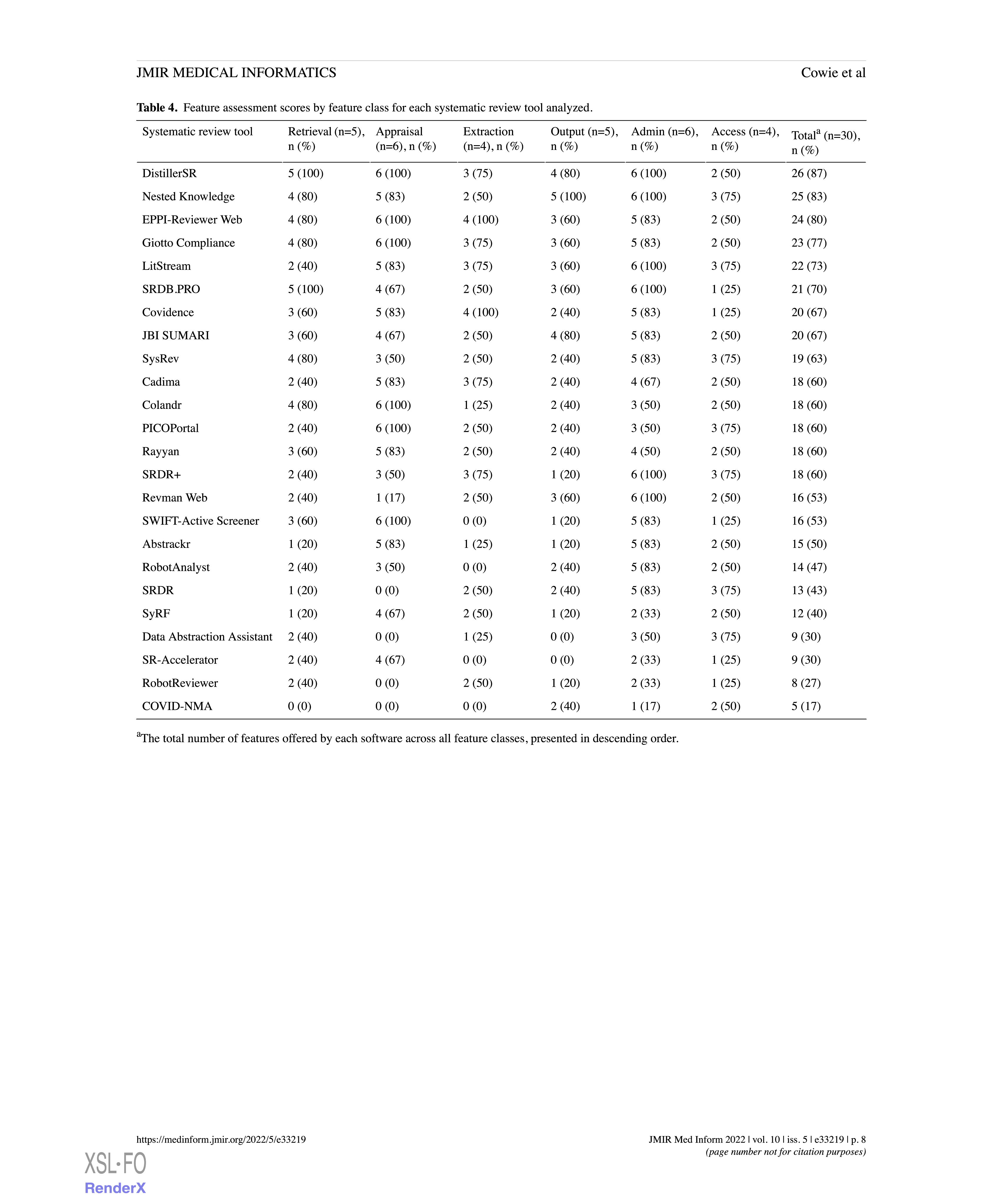

Supplement: Multimedia Appendix 1 [file medinform_v10i11e43520_app1.png]
